# Aggressive Progression of a WHO Grade I Meningioma of the Posterior Clinoid Process: An Illustration of the Risks Associated With Observation of Skull Base Meningiomas

**DOI:** 10.7759/cureus.14005

**Published:** 2021-03-19

**Authors:** Isabella M Young, Jacky Yeung, Chad Glenn, Charles Teo, Michael E Sughrue

**Affiliations:** 1 Centre for Minimally Invasive Neurosurgery, Prince of Wales Private Hospital, Sydney, AUS; 2 Department of Neurosurgery, University of Oklahoma Health Sciences Center, Oklahoma City, USA; 3 Nerosurgery, Prince of Wales Private Hospital, Sydney, AUS

**Keywords:** meningioma, progression, who, observation

## Abstract

Benign, small, and asymptomatic World Health Organization grade I meningiomas are usually managed expectantly with surveillance imaging with the assumption that they are predictably slowing growing. In this paper, we report the case of an incidentally discovered small, right-sided posterior clinoid meningioma in a 53-year-old female. The tumor was managed conservatively but an annual surveillance magnetic resonance imaging demonstrated that the meningioma had an unexpected significant growth impinging on the brainstem, requiring surgical resection and radiosurgery for residual tumor. Despite histopathological confirmation of a grade I meningioma, the tumor recurred significantly and incurred substantial neurological deficits, requiring further surgery and radiotherapy. This report illustrates the potential pitfall for expectant management of small meningiomas in anatomically precarious locations and draws attention to the need for detailed informed discussions with patients regarding the management of these tumors.

## Introduction

Meningiomas make up approximately 15-20% of all primary brain tumors [1,2]. Given that approximately 80% of meningiomas are considered “benign,” there is general agreement that not all of these tumors require treatment at initial diagnosis [2-4]. This has been especially argued in the petrous apex region due to difficult neurosurgical access and because it involves important neurological structures [5]. Similarly, radiotherapy confers non-trivial risks as well, given the proximity of this region to radiosensitive structures [6-9]. The option to observe rather than resect benign meningiomas has increased by 13% from 2004 to 2014, suggesting clinicians’ propensity for conservative management [10].

Traditional neurosurgical dictum mandates that one should not risk harm for something that may not require treatment. In meningioma treatment decision-making, there is a calculated risk inherent in the assumption that growth for a World Health Organization (WHO) grade I meningioma can be caught in time to intervene with a comparable risk. A contrasting example is glioblastoma, where we know that there is little chance the tumor will not progress if untreated, and that the amount of growth in an interval of observation will likely render treatment more dangerous, less possible, or even pointless.

Here, we present a case where despite the correct estimation that the tumor was benign, and a reasonable decision to obtain follow-up imaging on a small asymptomatic meningioma, the patient was left in a substantially worse position when the premises of that decision were found to be incorrect, in that the tumor expanded substantially and left the patient in a bad condition. While not typical, this case highlights that observation is not always safer than intervention, and therefore should be described as a calculated risk during surgical counseling with the patient.

## Case presentation

**Figure 1:**
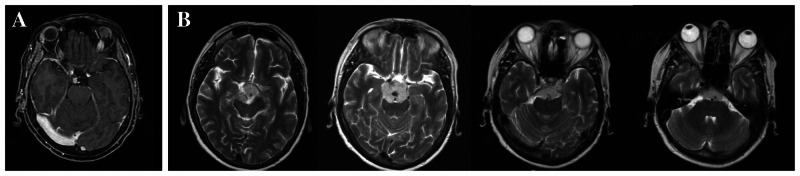
Pre-operative axial T2 imaging of the patient with a right-sided posterior clinoid meningioma. (A) Initial discovery of the tumor, which was observed yearly. (B) Substantial growth of the meningioma over a one-year period, wherein the tumor now extends along the clivus, encasing the basilar apex, and had started to compress the brainstem.

A 53-year-old woman presented to an outside hospital after an automobile accident where a brain magnetic resonance imaging (MRI) demonstrated a small, right-sided posterior clinoid meningioma (Figure 1A). After discussion with a neurosurgeon, the patient decided to observe this small, asymptomatic mass with yearly MRI scans.

**Figure 2:**
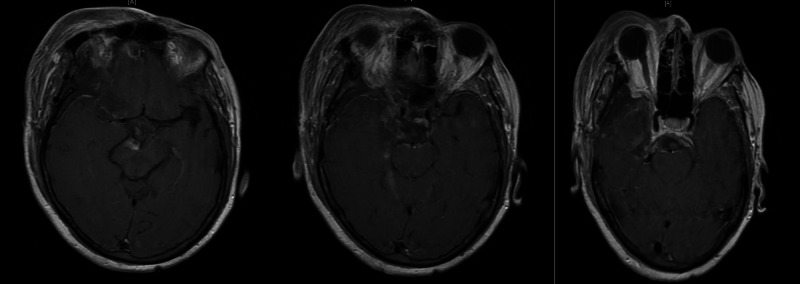
Post-operative axial T1 imaging of our patient with right-sided posterior clinoid meningioma following an orbitozygomatic approach with anterior, transpetrosal drilling. We achieved an extent of resection of 95%. A small, inferior portion was left behind due to adherence to the cranial nerves.

Upon her repeat imaging approximately one year later, MRI revealed that the tumor had grown substantially (Figure 1B). At this point, the tumor now extended along the clivus, was encasing the basilar apex, and was beginning to compress the brainstem. The case was referred to our center where the patient underwent an orbitozygomatic approach with anterior, transpetrosal drilling. We achieved an extent of resection of 95%, with a small, inferior portion left behind in an attempt to preserve the third nerve (Figure 2). Histopathology demonstrated a WHO grade I meningioma. The residual tumor was treated with gamma knife radiosurgery, dosed at 14 Gy to the 50% isodose line. At this point, the patient had mild third and fifth nerve palsies, but had normal visual acuity and was otherwise stable.

The patient was lost to follow-up for about two years. When she returned to our clinic, she had lost vision in her right eye. Imaging demonstrated substantial recurrence of her tumor, which was now extending along the clivus, invading both cavernous sinuses, and entering the sphenoid. She underwent surgery at another facility where an endonasal, endoscopic optic nerve decompression and sphenoidal tumor debulking were performed. This operation did not restore her vision in the affected eye. Histopathology at this time redemonstrated a WHO grade I meningioma. At this point, the patient underwent intensity-modulated radiation therapy as the tumor continued to show progressive skull base growth. At one year following radiotherapy treatment, the patient showed no signs of further tumor progression.

## Discussion

While this case is not reflective of the behavior of most histologically benign meningiomas, it does suggest that there is no true benign brain mass without the potential to grow [11]. It is possible to watch some small, asymptomatic meningiomas with acceptable outcomes. The National Comprehensive Cancer Network guidelines suggest observational imaging on unresected meningiomas every three months for the first year and then a biannual follow-up for the following five years [12]. It is important to note the limits of our predictive abilities and to acknowledge that population outcomes do not always reflect the individual needs of patients [13,14].

The principle assumption one makes when choosing not to treat a brain tumor is that there is a reasonable chance the patient will not need therapy at some point in their lives, and/or that if the tumor changes, there will be sufficient time to identify and intervene prior to the patient’s clinical status worsening. However, when a meningioma does become symptomatic, it is conceivable that the tumor is causing severe compression and irritation of neural elements and surgical intervention would confer higher risk.

While the literature is ambiguous, it seems reasonable that most people will eventually show some signs of tumor growth over their life expectancy [11,15]. While it does not follow that all small, asymptomatic tumors need immediate treatment, the idea that patients will be able to observe their tumor over their lifetime seems unlikely [4]. Put simply, most individuals will eventually require therapeutic intervention. In addition, a meningioma with TERT mutations harbors higher risk of malignant transformation and a more aggressive clinical course [16-18].

This case also demonstrates that the “watchful waiting” approach is not always optimal as even small tumors can cause neurologic decline. This is especially true for difficult tumors such as those in the petroclival region or the tuberculum sella, where we would like to avoid operating on asymptomatic patients if possible [19]. However, a small amount of tumor growth in these regions can substantially increase the risk of surgery and can lead to irreversible damage to the surrounding neural structures [20]. This is even more true in a case such as this in which the tumor grew aggressively.

## Conclusions

Given the medical community is trending towards conservative approaches to benign meningiomas, our goal is to point out that it’s not prudent to assume that observation is always the best plan. This paper does not claim that all asymptomatic meningiomas should undergo treatment. Rather, surgeons should have informed discussions with patients that it is not always the safest to observe the tumor, and it is not always the safest to ensure tumor growth before treatment.
